# The coat protein of citrus yellow vein clearing virus directly targets the ascorbate peroxidase 1 in lemon (ClAPX1) to facilitate virus accumulation

**DOI:** 10.3389/fpls.2023.1306580

**Published:** 2023-11-28

**Authors:** Chunqing Wang, Qi Zhang, Jiaxin Li, Xinliang Wang, Chuxin Li, Yu Bin, Zhen Song

**Affiliations:** ^1^ Citrus Research Institute, Southwest University, Chongqing, China; ^2^ Southwest University, Integrative Science Center of Germplasm Creation in Western China, Chongqing, China

**Keywords:** reactive oxygen species, ascorbate peroxidase, citrus yellow vein clearing virus, coat protein, jasmonic acid

## Abstract

Reactive oxygen species (ROS) are closely related to the antiviral immune response of plants, while virus can regulate ROS through various pathways to facilitate their own infection or replication. Citrus yellow vein clearing virus (CYVCV) is one of the most devastating viruses affecting lemon (*Citrus limon*) industry worldwide. However, the pathogenesis of CYVCV remains poorly understood. In this study, direct interaction between the coat protein (CP) of CYVCV and the ascorbate peroxidase 1 of lemon (ClAPX1) was confirmed for the first time by yeast two-hybrid, Bimolecular Fluorescence Complementation, and Co-immunoprecipitation assays. Transient expression of CP in lemon and *Nicotiana benthamiana* significantly enhanced the enzyme activity of the ClAPX1, and then inhibited the accumulation of H_2_O_2_. In addition, overexpression of ClAPX1 in lemon by transgene significantly promoted CYVCV accumulation and depressed the expression of most genes involved in jasmonic acid (JA) signaling pathway. Correspondingly, ClAPX1 silencing by RNA interference inhibited CYVCV accumulation and increased the expression of most genes involved in JA signaling pathway. To our knowledge, this is the first report that viruses regulate ROS by targeting APX directly, thereby suppressing host immune response and promoting viral accumulation, which may be mediated by JA signaling pathway.

## 1 Introduction

Reactive oxygen species (ROS), including superoxide (O_2_^-^), hydroxyl radicals (·OH), hydrogen peroxide (H_2_O_2_), is critical for plant development, response to abiotic and biotic stresses. Studies have shown that a local ROS burst can directly limit virus spread and ROS also act as signaling molecules to induce or trigger antivital immune responses, including pathogen-associated molecular pattern-triggered immunity (PTI), effector-triggered immunity (ETI), and systemic acquired resistance (SAR) (Durrant and Dong, 2004; Mittler, 2017; Li et al., 2021). On the other hand, virus can also promote their own infection or replication by regulating ROS via various pathways. The p27 protein of red clover necrotic mosaic virus (RCNMV) utilizes a respiratory burst oxidase homologue (RBOH) of *Nicotiana benthamiana* (*N. benthamiana*) to induce an intracellular ROS burst, which promoting viral RNA replication (Hyodo et al., 2017). The interaction between the helper component proteinase (HCPro) of chilli veinal mottle virus (ChiVMV) and the catalase (CAT) of *N. benthamiana*, induces a ROS burst to facilitate viral infection (Yang et al., 2020). In addition, the 2b protein of cucumber mosaic virus (CMV) directly interacted with CATs and inhibited their activities to facilitate ROS accumulation, thereby promotes its accumulation in host (Yang et al., 2022). The P31 protein of maize chlorotic mottle virus (MCMV) hijacks maize CAT1 resulting in H_2_O_2_ accumulation and enhanced viral multiplication. Further studies showed that P31 attenuated the expression of salicylic acid (SA)-responsive pathogenesis-related (PR) genes by inhibiting CAT activity during MCMV infection (Jiao et al., 2021). Barley stripe mosaic virus (BSMV) gb interacts with glycolate oxidase and inhibits peroxisomal ROS production to facilitate virus infection (Yang et al., 2018).

Ascorbate peroxidase (APX) is a key enzyme in ROS-scavenging system and plays a significant role in plant responding to abiotic and biotic stresses. APX distributed in various cellular compartment as cytoplasm, chloroplast and microbody, can be divided into four isoforms, such as: thylakoid membrane-bound APX (tAPX), microbody (including glyoxysome and peroxisome) membrane-bound APX (mAPX), stromal APX (sAPX), and cytosolic APX (cAPX) (Panchuk et al., 2005; Shigeoka et al., 2014). In abiotic stress, overexpression of the APX1 enhances ascorbate content and drought resistance in *Arabidopsis* (Liu et al., 2019). Overexpression of APX induces orchestrated ROS scavenging and enhances cold and heat tolerances in tobacco (Wang et al., 2017). Overexpression of thylakoid APX in tomatoes shows enhanced resistance to chilling stress (Duan et al., 2012). Stromal *OsAPX7* modulates drought stress tolerance in rice (*Oryza sativa*) (Jardim et al., 2023). In biotic stress, overexpression of the OsAPX8 increases tolerance to bacterial blight (Jiang et al., 2016). *Zea mays* APX1 overexpression resulted in lower H_2_O_2_ accumulation and enhanced resistance against *B. maydis* (Zhang et al., 2022). Tomato seedlings subject to 2,6-Dichloroisonicotinic acid (INA) treatment that significantly increase APX2 enzyme activity and enhanced resistance to *Xanthomonas perforans* (Chandrashekar and Umesha, 2014). Studies on citrus have also reported that APX enzyme activity remains at high levels after citrus tristeza virus infection of citrus (Pérez-Clemente et al., 2015). Mittler reported that tobacco mosaic virus infection did not affects the transcription of APX, but inhibits the translational extension of APX and down-regulates enzyme activity, leading to H_2_O_2_ accumulation and inducing an immune response in tobacco (Mittler et al., 1998). To date, the direct interaction between APX and protein of plant virus has not been reported.

Citrus yellow vein clearing virus (CYVCV) is an emerging virus that causes serious economic damage to the lemon industry worldwide. The virus was first identified in Pakistan in 1988 (Catara et al., 1993), and later spread rapidly into India, Turkey. In China, CYVCV was first identified in Eureka lemon (C. limon Burm. f.) in 2009 in Yunnan province (Chen et al., 2014), and spread rapidly in other citrus producing regions (Zhou et al., 2017). The virus is usually asymptomatic in most citrus species, cultivars and hybrids, but severe leaf distortion and yellow vein clearing are found in lemon (Citrus limon Burm. f.) and sour orange (C. aurantium L.) (Alshami et al., 2003; Önelge et al., 2007; Iftikhar et al., 2010; Loconsole et al., 2012). It caused particularly serious damage, resulting in reduced tree vigor, lower yields, and decreased marketability of fruit production. and it is easily transmitted by grafting, contaminated tools, Dialeurodes citri, and several aphid species (Zhang et al., 2018; Zhang, et al., 2019; Afloukou et al., 2021), which may further make the spread of the disease quick and extensive. Therefore, it is necessary to pay attention to the virus and exploit useful methods to control this virus.

CYVCV is a positive-sense single-stranded virus that belongs to genus *Mandarivirus* in the family *Alphafexiviridae* (Loconsole et al., 2012)The viral genome is about 7.5 kb, contains six predicted open reading frames (ORF) (Loconsole et al., 2012; Song et al., 2015). ORF1 encodes a putative RNA-dependent RNA polymerase (RdRp). ORF2-ORF4 encodes the triple gene block proteins (TGB1, TGB2, TGB3). ORF5 encodes the coat protein (CP), which considered to be a stronger RNA silencing suppressor (RSS), and associated with the pathogenicity of CYVCV (Rehmana et al., 2019; Bin et al., 2019). ORF6 encodes a 23 kDa protein of unknown function. Until now, the pathogenesis of CYVCV remains unclear. The CP of CYVCV can interact with its own TGB1, TGB3 and TGB triple gene block proteins, which may be involved in virus movement (Rehmana et al., 2019). Additionally, the CP exhibits a stronger RSS activity. Previous studies have demonstrated that plant viruses can utilize encoded RSS as pathogenic or symptomatic determinant (Ma et al., 2019). Additionally, the titer of CYVCV is positively associated with the severity of symptoms in infected citrus seedings (Bin et al., 2022). Furthermore, CP plays a crucial role in the infection and pathogenicity of CYVCV (Bin et al., 2019). However, there have been few studies on the interaction between CYVCV and host factors. Previously, a total of 32 host factors that interact with the CP of CYVCV were initially screened from the cDNA library of Eureka lemon by Bin Yu et al. (Bin et al., 2023a); however, the precise mechanism underlying these interactions remains unknown. A previous study has reported that the activity of APX1 enzyme increased significantly in CYVCV-infected lemons,ClAPX1 was identified as a cellular protein (Zhang et al., 2022), and the APX that interact with CP of CYVCV was initially identified by Bin Yu et al. (Bin et al., 2023a), but its function and mechanism still need to be clarified. Therefore, it is essential to explore the interaction between CYVCV and host factors, which may provide efficient strategies for disease control.

In this study, in order to clarify the pathogenesis of CYVCV, explore the interaction between CYVCV and host factors, to exploit resistant varieties for effective virus control, we confirm that there is a direct interaction between CP of CYVCV and ClAPX1 for the first time. The transient expression of CP promoted the enzyme activity of ClAPX1 and reduced the accumulation of H_2_O_2._ In addition, overexpression of ClAPX1 facilitates the CYVCV accumulation and inhibited the expression of most genes associated with JA signaling pathway, the accumulation of CYVCV was reduced and the expression level of most genes involved with JA signaling pathway were elevated in ClAPX1-silenced plants.

## 2 Materials and methods

### 2.1 Plant material and growth conditions

A CYVCV infectious cDNA clone, pCY-AY221, was constructed by Cui et al. (Cui et al., 2018). *N. benthamiana* were grown in a greenhouse at 25°C under a 16 h/8 h light/dark cycle. Eureka lemon (*Citrus limon (L.) Osbeck*) seeds were maintained in a greenhouse at 25°C under a 16 h/8 h light/dark cycle.

### 2.2 Yeast two-hybrid assay

The cDNA library was constructed by the Genecreate company using Eureka lemon leaves infected with CYVCV. The coding sequence (CDS) of the CP was cloned into the pGBKT7 vector to generate the pGBKT7-CP vector. Subsequently, The CDS of ClAPX1 was cloned and fused with the pGADT7 vector to generate the pGADT7-ClAPX1 vector. The generated bait and prey plasmids were co-transformed into yeast cells (Y2H Gold) and cultured on SD-Leu/-Trp medium for 2-3 days post inoculation (dpi). Then, they were plated onto SD-Leu/-Trp/-His medium to analyze the interaction. The primers used for vector construction are listed in **Supplementary Table S1**.

### 2.3 Bimolecular fluorescence complementation

The CDS of CP and ClAPX1 were inserted into the pSPYNE and pSPYCE vectors, respectively, to generate the pSPYNE-CP and pSPYCE-ClAPX1 constructs. The obtained vectors were transformed into *A. tumefaciens* strain GV3101 and cultured overnight. The cultures were collected and resuspended with infiltration buffer (10 mM MES, 10 mM MgCl_2_, 200 μM acetosyringone) with OD_600 = _1.0. *Agrobacterium* strain GV3101 with pSPYNE-CP and pSPYCE-ClAPX1 were mixed (1:1 ratio), followed by incubation at 25°C for 3 h. Subsequently, the mixture was infiltrated into *N. benthamiana* leaves. The infiltrated leaves were collected using a 5 mm diameter punch and observed under an Olympus FV3000 confocal microscope at 2-3 dpi. The primers used for vector construction are listed in **Supplementary Table S1**.

### 2.4 Co-immunoprecipitation assay

The CDS of CP and ClAPX1 were cloned into the pBI121 vector to generate pBI121-CP-RFP and pBI121-ClAPX1-CFP, respectively. *A. tumefaciens* harboring pBI121-CP-RFP and pBI121-ClAPX1-CFP were co-agroinfiltrated in *N. benthamiana* leaves. The infiltrated leaves were harvested at 2 dpi, frozen with liquid nitrogen, and subsequently ground into a powder. Total protein was then extracted using IP buffer (0.15 M NaCl, 0.05 M Tris-HCl, pH 7.5, 1 mM EDTA, 0.1% (v/v) Triton X-100, 10% (v/v) glycerol, 1×protease inhibitor cocktail and 1 mM phenylmethylsulfonyl fluoride (PMSF)). After being incubated for 20 min, the mixture was centrifuged at 12000 g, 4°C for 20 min. The supernatant was then incubated with anti-GFP beads. The immunoprecipitated proteins were separated by 12.5% SDS-PAGE and analyzed using the corresponding antibodies. The primers used for vector construction are listed in **Supplementary Table S1**.

### 2.5 Subcellular localization in *N. benthamiana*

The pBI121-CP-RFP and pBI121-ClAPX1-CFP vectors were used for the subcellular localization assay, and pBI121-CFP was used for control. The pBI121-ClAPX1-CFP construct was subsequently co-infiltrated into the leaves of *N. benthamiana* along with agrobacterial cells harboring the plasma membrane marker PM-GFP and the nuclear marker H2B-GFP. pBI121-CP-RFP and pBI121-ClAPX1-CFP were also co-infiltrated into *N. benthamiana* leaves. Green fluorescence protein (GFP), blue fluorescent protein (BFP) and red fluorescence (RFP) were observed using confocal laser scanning microscope FV3000 at 2-3 dpi.

### 2.6 Transient expression in *N. benthamiana* and citrus

*A. tumefaciens* GV3101, which harbors the pBI121-CP-RFP and pBI121-ClAPX1-CFP plasmids, were mixed in a 1:1 ratio for co-infiltration into *N. benthamiana* and one-year-old virus-free Eureka lemon seedlings, respectively. At 2-3 dpi, the infiltrated leaves of *N. benthamiana* were collected for CYVCV accumulation and additional analysis. ​At 10 and 20 dpi, the infiltrated lemon leaves were collected for observation of CYVCV accumulation and further analysis.

### 2.7 Citrus hairy root transformation

Citrus hairy root transformation was performed according to the methods reported by Ma et al. (2022). The CDS of ClAPX1 was amplified and inserted into the plant binary expression vector pLGN, which is driven by a 35S promoter. A fragment of about 400 bp from ClAPX1 was chosen as the target for silencing and cloned into the RNAi vector to generate the pLGN-RNAi-ClAPX1 vector. The plasmids were then transformed into *A. rhizogenes* strain K599 and cultured overnight at 28°C using TY liquid medium. The resuspended cell at final concentration (OD600 = 0.6-0.8) with the MES buffer (10 mM MgCl2, 10 mM MES [pH 5.6], and 200 µM AS) followed by incubated at 28°C for 3 h. Subsequently, Eureka lemon branches were soaked in the resuspended *A. rhizogenes* strains K599 and subjected to vacuum inﬁltration for approximately 25 min using a standard vacuum. The stem sections were cultured in a dome tray filled with vermiculite-mixed soil in the greenhouse at 26°C, with 90% relative humidity and a 16 h/8 h (light/dark) photoperiod. To identify the transgenic roots, GUS histochemical staining and reverse transcription-PCR (RT-PCR) assays were used. The obtained transgenic roots were used for gene expression analysis and subsequent evaluation. The primers used for vector construction are listed in **Supplementary Table S1**.

### 2.8 Tolerance of transgenic and silenced Eureka lemons to CYVCV

Transgene and silencing citrus were graft inoculated with CYVCV positive bark by RT-qPCR detection. Subsequently, root samples were collected one month after virus inoculation for gene expression and CYVCV accumulation analysis. The primers used for vector construction are listed in **Supplementary Table S1**.

### 2.9 Western blot

The total protein of *N. benthamiana* leaves, Eureka lemon leaves and roots were extracted according to Plant Total Protein Extraction Kit (Solarbio Life Sciences). The extracted proteins were then separated using 10% SDS-PAGE and then transferred to the PVDF membrane. The PVDF membranes were incubated with monoclonal anti-CP of CYVCV, monoclonal anti-GFP (Sigma), followed by a horseradish peroxidase (HRP)-conjugated secondary antibody (Proteintech). Blots were visualized using a chemiluminescence detection kit (Everbright Inc.).

### 2.10 Biochemical analysis

The hydrogen peroxide (H_2_O_2)_ contents were measured using the micro method kit following the manufacturer’s instructions (Nanjing molfarming Biotech Co., Ltd., Nanjing, China). Three biological replicates were maintained in each group. The ClAPX1 activity was measured using a micro method kit following the manufacturer’s instructions (Beijing Solarbio Science & Technology Co., Ltd., Beijing, China).

### 2.11 Total RNA extraction and RT-qPCR analysis

The total RNA was isolated from *N. benthamiana* or citrus leaves or roots using the RNAiso plus, 1st Strand cDNA Synthesis SuperMix (Novoprotein, Japan) was used to generate the first-strand cDNA. RT-qPCR was conducted using BlasTaq™ 2 × qPCR mixes, *N. benthamiana* and citrus actin gene were used as an internal reference. The results were analyzed by the 2^-ΔΔCt^ method and shown as means ± SD (n = 3). At least three biological repeats were conducted for all experiments. Different letters indicate significant differences at P ≤ 0.05.

### 2.12 Statistical analysis

Each experiment was repeated at least three times, and data are represented as the mean. Values are presented as means ± SD. Statistical significance was determined using Student’s t-test, * P < 0.05; ** P<0.01, *** P<0.001. All analyses were performed with the GraphPad Prism 8.0 software.

## 3 Results

### 3.1 CP of CYVCV interacts with ClAPX1 both *in vivo* and *in vitro*

**Figure 1:**
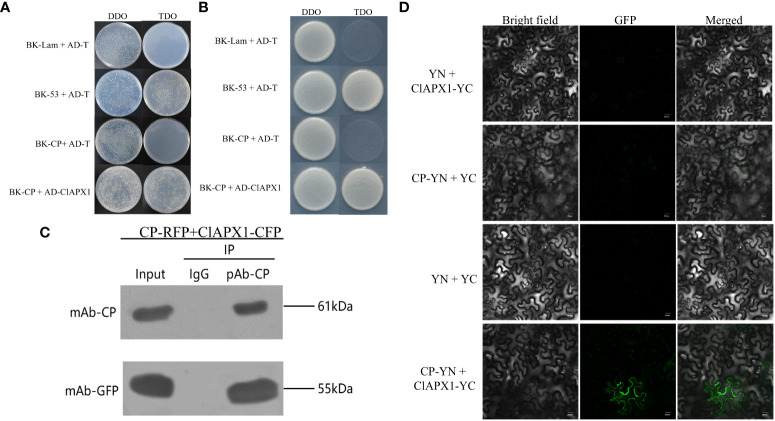
The coat protein (CP) of citrus yellow vein clearing virus (CYVCV) directly interacts with Ascorbate peroxidase 1 of lemon (ClAPX1) *in vitro* and *in vivo*. **(A–D)**. **(A, B)** Yeast two-hybrid (Y2H) analysis. Plasmid combinations are indicated on the left. **(A)** represent cells of yeast strain Y2H Gold co-expressing the indicated plasmid combinations were spotted onto yeast synthetic defined (SD) medium SD/-Trp/-Leu and SD/-Trp/-Leu/-His. The yeast cells harboring BK-CP + AD-T, and BK-Lam + AD-T were used as negative controls, and BK-53 + AD-T was used as positive control. **(B)** represent a single clone was select from **(A)** and plate on to the (SD) medium SD/-Trp/-Leu and SD/-Trp/-Leu/-His. **(C)** Coimmunoprecipitation (co-IP) assay. The pBI121-CP-RFP construct was transiently co-expressed with pBI121-ClAPX1-CFP in *Nicotiana benthamiana* (*N. benthamiana*) leaves by agroinfiltration, total protein was extracted at 3 days post inoculation (dpi) and immunoprecipitated with anti-CP and anti-GFP beads. Input and IP immunoprecipitation were analyzed by immunoblotting with anti-GFP or anti-CP antibodies. **(D)** Bimolecular fluorescence complementation (BiFC) assay, the experiments was conduncted on plants epidermis tissue. Plasmid combinations were co-infiltrated into *N. benthamiana* leaves; GFP fluorescence was visualized by confocal microscopy at 2 dpi. The Green fluorescence indicates an interaction between CP and ClAPX1. Scale bars=20 μm.

In order to verify the direct interaction between CP and ClAPX1, Y2H, BiFC, and CO-IP assays were conducted. The Y2H assay demonstrated that PGBKT7-CP and PGADT-ClAPX1 exhibited natural growth on the SD/−Trp/−Leu/−His (TDO) medium, while the negative control did not show any growth (**Figures 1A, B**), indicating a direct interaction between CP and ClAPX1. To further confirm this interaction, BiFC assay was conducted on plants epidermis tissue. At 2 dpi, GFP was observed in the plasma membrane of *N. benthamiana* co-infiltrated with *Agrobacterium* carrying pSPYNE-CP and pSPYCE-ClAPX1 plasmids, but not in those co-infiltrated with pSPYNE-CP and pSPYNE or pSPYCE-ClAPX1 and pSPYCE plasmids (**Figure 1D**). These findings provide evidence that CP interacts with ClAPX1 in the plasma membrane *in vivo*. Additionally, Co-IP results also confirmed the interaction between CP and ClAPX1 (**Figure 1C**). Taken together, these results unequivocally establish that CP of CYVCV directly interacts with ClAPX1 both *in vivo* and *in vitro*.

### 3.2 Subcellular location of CP of CYVCV and ClAPX1

**Figure 2:**
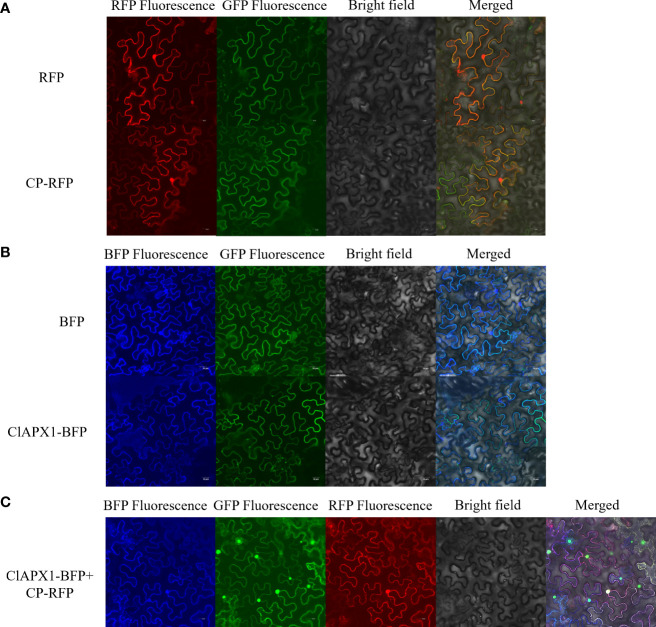
Subcellular localization of CP of CYVCV and ClAPX1 in *N. benthamiana* leaves. **(A–C)**. **(A)** CP of CYVCV fused with RFP and empty RFP-containing vectors were transiently expressed. **(B)** ClAPX1 fused with BFP and empty BFP-containing vectors were transiently expressed. **(C)** CP fused with RFP and ClAPX1 fused with BFP were transiently co-expressed. Scale bars=20 μm. the experiments was conduncted on plants epidermis tissue.

The subcellular location of CP and ClAPX1 were investigated in epidermis tissue of *N. benthamiana*. When expressed individually, CP was found to be localized in the nucleus and the plasma membrane (**Figure 2A**), while ClAPX1 was localized solely in the plasma membrane (**Figure 2B**). In contrast, co-expression of both proteins resulted in their localization on the plasma membrane (**Figure 2C**). These findings further support the interaction between CP and ClAPX1 on the plasma membrane as demonstrated by BiFC analysis.

### 3.3 CP enhanced the H_2_O_2_ scavenging activity of ClAPX1

**Figure 3:**
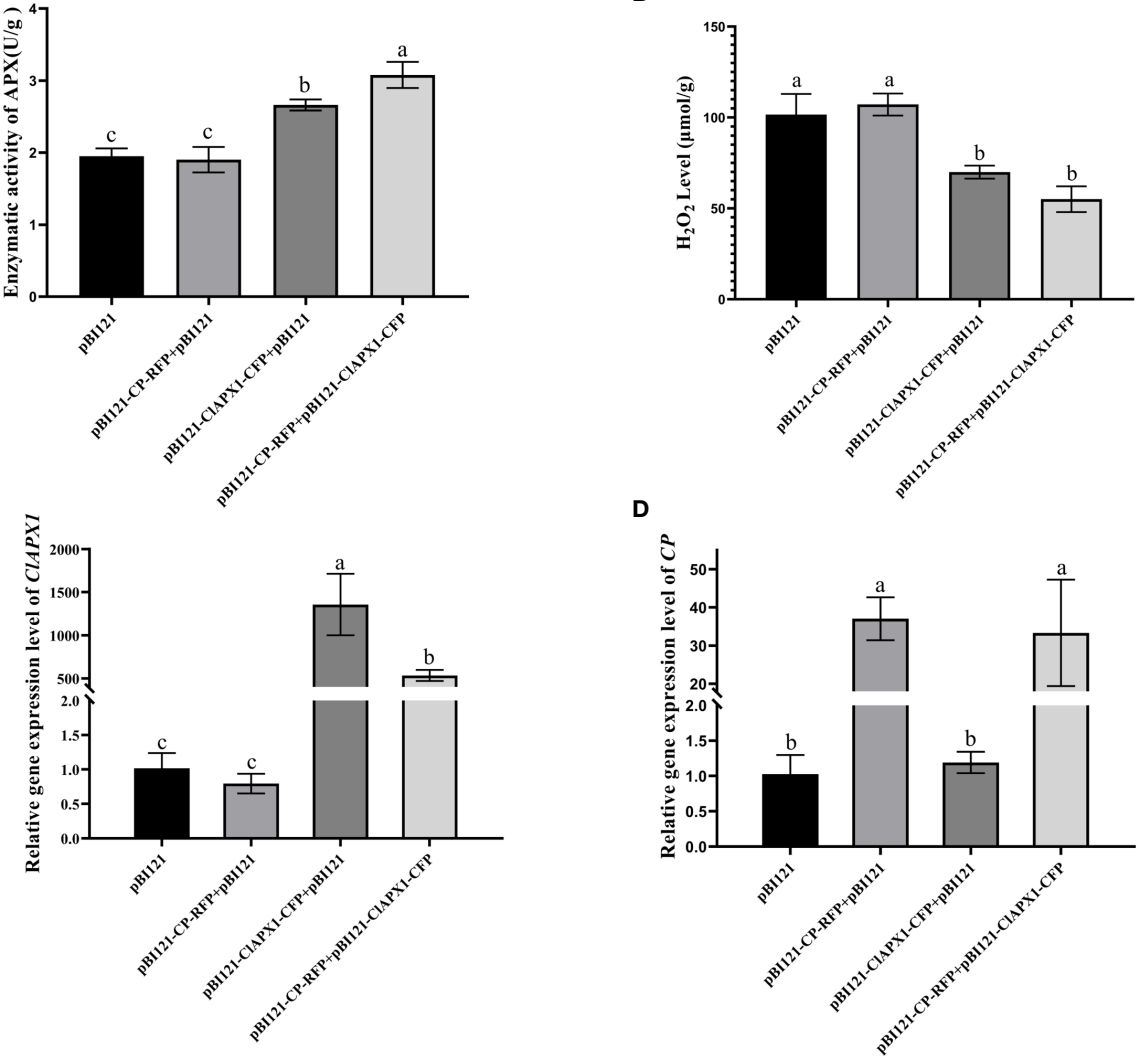
CP enhanced the H_2_O_2_ scavenging activity of ClAPX1 through transient expression CP and ClAPX1 in *N. benthamiana* leaves at 3 dpi. **(A–D)** The CP and ClAPX1 were cloned into the pBI121 vector to generate pBI121-CP-RFP and pBI121-ClAPX1-CFP for transient expression, respectively. The three groups pBI121-CP-RFP+pBI121, pBI121-ClAPX1-CFP+pBI121, pBI121-CP-RFP+pBI121-ClAPX1-CFP represented *A*. *tumefaciens* harboring pBI121-CP-RFP mixed with pBI121, pBI121-ClAPX1-CFP mixed with pBI121, pBI121-CP-RFP mixed with pBI121-ClAPX1-CFP were co-agroinfiltrated in *N. benthamiana* leaves, respectively. The pBI121 was used as the control. **(A)** Quantification of ClAPX1 enzyme activity. **(B)** Quantification of H_2_O_2_ accumulation. **(C)** the relative expression level of *ClAPX1* gene. **(D)** the relative expression level of CP of CYVCV. Error bars represented standard error of the means, letters represent significant differences between samples, one-way ANOVA with a p-value of 0.05 by least significant method (LSD) *post hoc* test was performed.

To investigate the biological function of the interaction between CP and ClAPX1, both proteins were transiently expressed individually and co-expressed on *N. benthamiana* through *Agrobacterium* mediated injection. At 3 dpi, the enzyme activity of ClAPX1 and the H_2_O_2_ content of the inoculated leaves were measured. The findings revealed a significant increase in enzyme activity and a significant decrease in H_2_O_2_ content in leaves expressing ClAPX1, while no changes were observed in leaves expressing CP (**Figures 3A, B**). These results suggest that ClAPX1 possesses the ability to scavenge ROS.

Furthermore, compared to expression of ClAPX1 alone, the co-expression of CP and ClAPX1 resulted in a significant increase in enzyme activity and a decrease in H_2_O_2_ content, as observed in **Figures 3A, B**. This suggests that CP has a positive effect on enhancing the enzyme activity of ClAPX1. Additionally, the transcriptional analysis confirmed the expression of CP and ClAPX1, indicating that CP negatively regulates the expression of ClAPX1, while ClAPX1 does not affect the expression of CP, as shown in **Figures 3C, D**. Overall, these findings suggest that CP likely enhances the H_2_O_2_ scavenging activity of ClAPX1 through direct interaction.

### 3.4 Transient expression of ClAPX1 enhanced CYVCV accumulation in *N. benthamiana* and citrus

**Figure 4:**
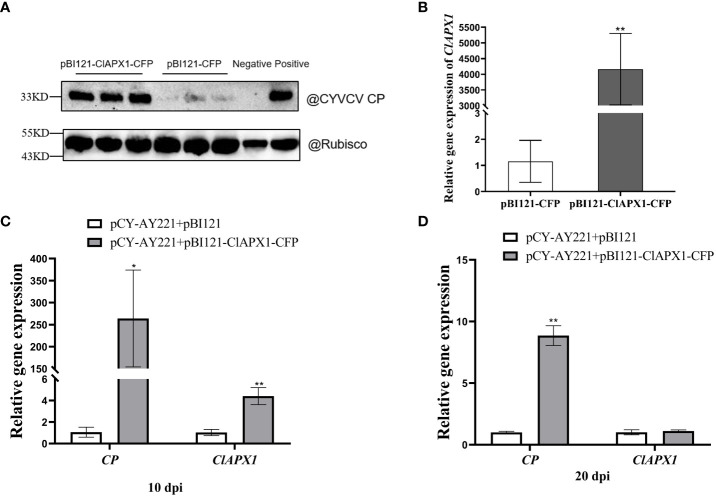
Transient expression of ClAPX1 facilitate CYVCV accumulation in *N. benthamiana* and lemon. **(A–D)** The ClAPX1 was cloned into the pBI121-CFP to generated the pBI121-ClAPX1-CFP for the transient expression. The pBI121-CP-RFP and pBI121-ClAPX1-CFP were co-infiltrated into the *N. benthamiana.* The pBI121 vector was used as a control. **(A)** quantification of CYVCV accumulation by western blot analysis after transient ClAPX1 expression in *N. benthamiana* at 3dpi. Rubisco was used as a loading control. **(B)** the relative expression level of *ClAPX1* gene after transient ClAPX1 expression in *N. benthamiana* at 3dpi. The pCY-AY221, a CYVCV infectious clone, pBI121-ClAPX1-CFP were co-infiltrated into *N. benthamiana*. **(C)** and **(D)** the relative expression level of *ClAPX1* and CYVCV accumulation after transient expression in lemon at 10 dpi and 20 dpi. Bars represent standard deviation values, statistical significance was determined using Student’s t-test (* *P* < 0.05, ** *P* < 0.01).

To investigate the impact of ClAPX1 on CYVCV accumulation, *Agrobacterium* carrying pBI121-ClAPX1-CFP or pCY-AY221, an infectious clone of CYVCV, were co-infiltrated into *N. benthamiana* plants. At 3 dpi, the expression level of the *ClAPX1* gene and the titer of CYVCV were quantified using RT-qPCR and western blotting, respectively. The results demonstrated a significant increase in CYVCV titer (**Figure 4A**) upon overexpression of *ClAPX1* gene (**Figure 4B**), indicating that elevated levels of ClAPX1 enhance CYVCV accumulation. Additionally, a similar experiment was conducted on lemon leaves. At 10 dpi, both the expression level of *ClAPX1* and the titer of CYVCV showed significant increases compared to control samples (**Figure 4C**). Even at 20 dpi, there was still higher accumulation level of CYVCV compared to controls (**Figure 4D**). These findings highlight that transient expression of ClAPX1 promotes accumulation of CYVCV.

### 3.5 Over-expression of ClAPX1 enhanced the accumulation of CYVCV in transgenic lemon while ClAPX1 silencing depressed the virus

To further elucidate the role of ClAPX1 in CYVCV accumulation, transgenic lemon lines overexpressing and silencing ClAPX1 in roots were generated and subsequently subjected to CYVCV inoculation via grafting. At 30 dpi, samples were collected from the infected roots for subsequent analysis using RT-qPCR and western blotting techniques.

**Figure 5:**
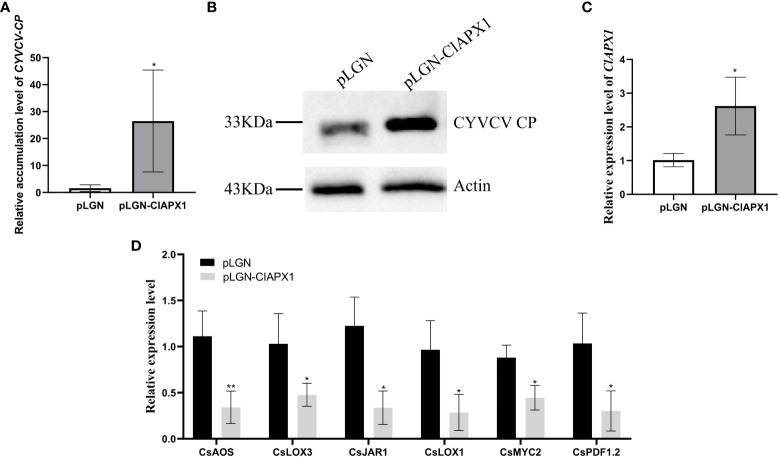
Overexpression of ClAPX1 facilitates CYVCV accumulation in transgenic lemon. **(A–D)**. **(A)** The relative accumulation level of CYVCV in transgenic citrus roots in mRNA level. **(B)** the accumulation level of CYVCV in transgenic citrus roots by western blot analysis, Actin served as loading control. **(C)** the relative expression level of *ClAPX1* gene in transgenic citrus roots. **(D)** the relative transcript levels of the jasmonic acid (JA) biosynthesis and responsive genes in the transgene citrus roots. Bars represent standard deviation values, statistical significance was determined using Student’s t-test (* *P* < 0.05, ** *P* < 0.01).

**Figure 6:**
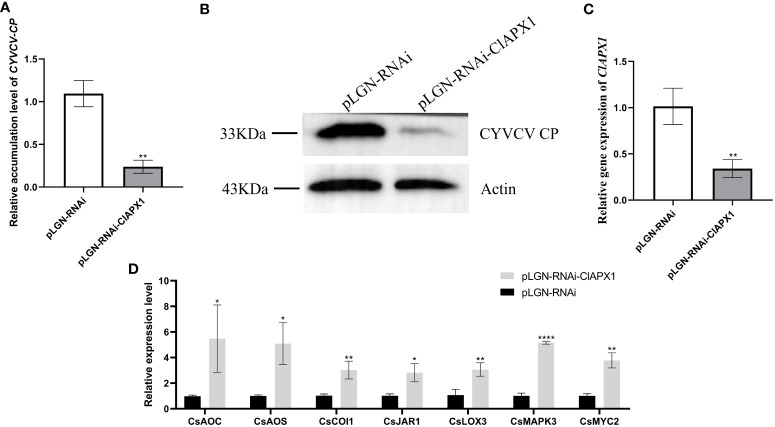
Silence of *ClAPX1* inhibits CYVCV accumulation in silenced citrus. **(A–D)**. **(A)** The relative accumulation level of CYVCV in silenced citrus roots by RT-qPCR. **(B)** the accumulation level of CYVCV in silenced citrus roots by western blot analysis, Actin served as loading control. **(C)** the relative expression level of *ClAPX1* gene in silenced citrus roots. **(D)** relative transcript levels of JA biosynthesis and responsive genes in silenced citrus roots. Bars represent standard deviation values, statistical significance was determined using Student’s t-test (* *P* < 0.05, ** *P* < 0.01, **** *P* < 0.0001).

The results revealed a significant increased levels of both CP mRNA and protein (**Figures 5A, B**) in roots with *ClAPX1* over-expression (**Figure 5C**) compared to the empty vector control. Conversely, silencing *ClAPX1* resulted in a significant downregulation of both CP mRNA and protein levels in roots (**Figures 6A–C**). These findings provide compelling evidence that the enhanced expression of ClAPX1 promotes increased accumulation of CYVCV in transgenic lemon plants, while silence of ClAPX1 restrains accumulation of CYVCV.

### 3.6 ClAPX1 negatively regulates genes involved in the JA signaling pathway during CYVCV infection

To elucidate the mechanism underlying ClAPX1-mediated facilitation of CYVCV accumulation, we examined the relative expression levels of ten genes involved in jasmonic acid (JA) biosynthesis and immune response, including allene oxide synthase (*CsAOS*), allene oxide cyclase (*CsAOC*), coronatine insensitive 1 (*CsCOI1*), lipoxygenase 3 (CsLOX3), lipoxygenase 1 (CsLOX1), jasmonic acid-amido synthetase 1 (CsJAR1), myelocytomatosis proteins 2 (CsMYC2), mitogen-activated protein kinase (*CsMAPKs*), plant defensin 1.2 (*CsPDF1.2*), and pathogenesis-related protein 3 (*CsPR3*) in transgenic lemon roots.

The results revealed that the expression levels of six genes, namely *CsAOS*, *CsLOX3*, *CsJARl*, *CsLOX1*, *CsMYC2*, and *CsPDF1.2*, significantly downregulated in ClAPX1-overexpressing plants (**Figure 5D**). Conversely, the expression levels of seven genes were significantly upregulated in ClAPX1-silenced roots, including *CsAOS*, *CsAOC*, *CsCOI1*, *CsJAR1*, *CsLOX3*, *CsMAPKs* and *CsMYC2* (**Figure 6D**). Thus, ClAPX1 negatively regulates genes involved in the JA signaling pathway during CYVCV infection. This may lead to a suppression of the immune response that facilitates the accumulation of the virus.

## 4 Discussion

**Figure 7:**
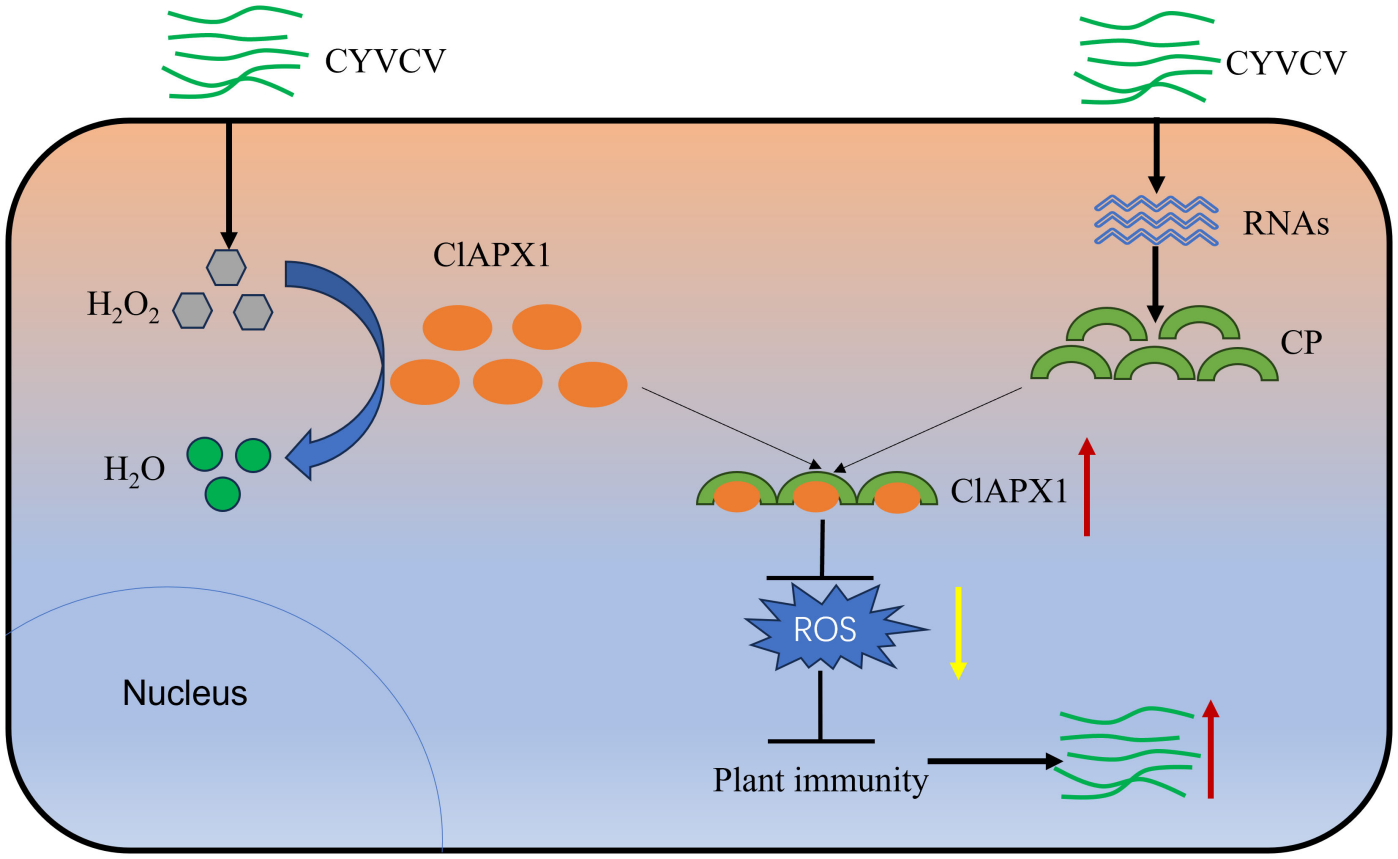
Proposed model. In plants, APX plays crucial roles in H_2_O_2_ scavenging and ROS homeostasis maintaining. When CYVCV infection, the CP directly targets ClAPX1 to promotes its enzyme activity and reduce ROS content in plant, thereby inhibiting the immunity of host plants and facilitate CYVCV accumulation, which may be mediated by JA signaling pathway.

ROS plays a crucial role in plant defense responses. However, plant viruses interfere with host ROS signaling to evade plant’s immunity response and promote virus infection or replication. A previous study has shown a significant increase in the H_2_O_2_ content in lemons infected with CYVCV (Zhang et al., 2023), suggesting a competitive relationship between the virus and ROS. However, the underlying mechanism and function remain unknown. In this study, it was found that CP of CYVCV directly targets the host protein ClAPX1, thereby enhancing its ROS scavenging activity in plants. This subsequently suppresses the host immune response and facilitates viral accumulation, potentially through modulation of the JA signaling pathway (**Figure 7**).

APX plays a major role in scavenging ROS. It is involved in the response to various stresses, including high light, drought, heat, bacterial and fungal infection (Chin et al., 2014; Bonifacio et al., 2016; Jiang et al., 2016; Du et al., 2023). However, the effects of APX in response to plant viruses remain largely unknown. The previous study has reported that the relative expression level of ClAPX1 and enzyme activity of CIAPX1 were higher when induced by the CYVCV-infected Eureka lemons (Zhang et al., 2023). These findings suggested that ClAPX1 may play a role in modulating the host-virus interaction. In this study, we demonstrate that the direct interaction between CP and ClAPX1 both *in vitro* and *in vivo*, thereby enhancing its activities and mitigating ROS accumulation. This study represents the first report to elucidate the direct interaction between CP and ClAPX1 in regulating ROS, thereby establishing a fundamental basis for unraveling the pathogenesis of CYVCV.

ROS burst is a plant defense response that limits the spread of viruses. To evade the immune mechanism of plants, viruses use their encoded protein to regulate ROS production or accumulation in order to manipulate host factors and promote infection. The interaction between Triple gene block protein 1 (TGBp1) of Pepino mosaic virus (PepMV) and tomato CAT1 increases its activity and promotes virus accumulation (Mathioudakis et al., 2013). In this study, we have demonstrated that transient expression and overexpression of ClAPX1 in citrus promote the CYVCV accumulation, while silencing ClAPX1 in citrus decreases CYVCV accumulation. This finding suggest that ClAPX1 may be a susceptible gene for CYVCV infection. A study has reported that engineering canker-resistant plants through CRISPR/Cas9-targeted editing of the susceptibility gene *CsLOB1* promoter in citrus (Peng et al., 2017), suggesting that it is possible to mutate or silence the APX gene either using the CRISPR-Cas9 system or RNAi system, thereby exploring resistant varieties for effective virus control.

The role of APX in pathogen infection has been extensively studied; however, the underlying mechanism by which APX responds to biological stress remains elusive. One study revealed that the cytosolic localized protein ZmAPX1 induces resistance to SCLB in maize by reducing H_2_O_2_ accumulation and activating a JA-mediated defense signaling pathway (Zhang et al., 2022). CAT, which has the same effect as APX, was inhibited during viral infection, and ROS species in plants were increased, while the expression of SA and PR pathway genes were reduced. Consequently, plant defenses were weakened and viral protein accumulation were enhanced (Jiao et al., 2021). In this study, we found that the interaction between CP and ClAPX1 enhances the enzyme activity of the ClAPX1, promoting CYVCV accumulation. However, the mechanism underlying plant susceptibility remains unclear. In a previous study, we found that the JA contents decreased, implying that JA maybe participate in the process of CYVCV infection (Bin et al., 2023b). In order to investigate the impact of ClAPX1 on the JA signaling pathway, the expression levels of ten JA biosynthesis and response genes were analyzed in transgenic and silenced citrus roots. It was found that the expression level of six genes (*AOS, LOX3, JAR1, LOX1, MYC2, PDF1.2*) were decreased in the transgenic roots, while the expression levels of seven genes (*AOS, LOX3, JAR1, MYC2, AOC, COI1, MAPK3*) were increased in the silenced roots. These results suggest that the interaction of CP with ClAPX1 may affect the JA signaling pathway, while further studies are needed to elucidate the mechanism by which APX promotes CYVCV accumulation.

In summary, our study confirmed that the CP of CYVCV can directly interact with the host protein ClAPX1, thereby promoting its activity and reducing ROS accumulation. Furthermore, the interaction between CP and ClAPX1 suppresses plant immunity, thereby facilitating CYVCV accumulation, which may be involved with JA signaling pathway. The present study represents the first report to clarify the direct interaction of CP with ClAPX1 via regulating ROS. This finding lays a foundation for future investigations aimed at unraveling the functional role of APX in plant-pathogen interactions and exploring potential resistant varieties.

## Data availability statement

The raw data supporting the conclusions of this article will be made available by the authors, without undue reservation.

## Author contributions

CW: Investigation, Validation, Writing – original draft, Writing – review & editing. QZ: Investigation, Writing – review & editing. JL: Writing – review & editing. XW: Writing – review & editing. CL: Writing – review & editing. YB: Writing – review & editing. ZS: Supervision, Writing – review & editing.
