# Supplementary material for: The coat protein of citrus yellow vein clearing virus directly targets the ascorbate peroxidase 1 in lemon (ClAPX1) to facilitate virus accumulation
Source: Front Plant Sci. 2023 Nov 28;14:1306580. doi: 10.3389/fpls.2023.1306580 (PMC10718646; doi:10.3389/fpls.2023.1306580)
Supplement: Supplementary file 1 [file Table_1.docx]

**Supplementary Table 1 |** Primers used in this study

| **Primer name** | **Sequence (5’-3’)** | **Application** |
| --- | --- | --- |
| BK-CP-F | TGATCTCAGAGGAGGACCTGCATATGAGCTTCGACTACACTCACCC | Construction of pGBKT7-CP vector |
| BK-CP-R | TGCGGCCGCTGCAGGTCGACGGATCCTTAGATGTTGAAAGGGGTC |  |
| AD-APX1-F | CCCGGGTGGGCATCGATACGGGATCCATGACGAAGAATTACCCCAC | Construction of pGADT7-ClAPX1 vector |
| AD-APX1-R | TATCTACGATTCATCTGCAGCTCGAGTTAGGCTTCAGCAAATCCTAG |  |
| pSPYNE-CP-F | CTCGAGGGTACCGCTCCCGGGATGAGCTTCGACTACACTCACC | Construction of pSPYNE-CP vector |
| pSPYNE-CP-R | CAACTTTTGCTCCATCCCGGGGATGTTGAAAGGGGTCGGGC |  |
| pSPYCE-ClAPX1-F | CTCGAGGGTACCGCTCCCGGGATGACGAAGAATTACCCCACTG | Construction of pSPYCE-ClAPX1 vector |
| pSPYCE-ClAPX1-R | ATCGTATGGGTACATCCCGGGGGCTTCAGCAAATCCTAGCTC |  |
| pBI121-ClAPX1-CFP-F | GAGAACACGGGGGACTCTAGAATGACGAAGAATTACCCCACTG | Construction of pBI121-ClAPX1-CFP vector |
| pBI121-ClAPX1-CFP-R | CCTCGCCCTTGCTCACCATGGTACCGGCTTCAGCAAATCCTAGCT |  |
| pBI121-CP-RFP-F | GAGAACACGGGGGACTCTAGAATGAGCTTCGACTACACTCACC | Construction of pBI121-CP-RFP vector |
| pBI121-CP-RFP-R | GAAGGCCATCTCGAGTCTAGAGATGTTGAAAGGGGTCGGGC |  |
| pLGN-6xHis-3-APX-F | gaggacagggtacccATGcatcatcatcatcatcacATGACGAAGAATTACCCCAC | Construction of transgene vector of pLGN-ClAPX1 |
| pLGN-6xHis-3-APX-R | cactagtggatccccTTAGGCTTCAGCAAATCCTAG |  |
| 3-APX-qF | GGCTTCCTGATGCTAAAC | Detection of *ClAPX* gene relative expression level by RT-qPCR |
| 3-APX-qR | CAATATCCTTGTCGCTGAG |  |
| pLGN-RNAi-3-APX-F1 | gatgatatcccatggggcgcgccAGCGACAAGGATATTGTTGC | Construction of RNAi vector of pLGN-RNAi-ClAPX1 |
| pLGN-RNAi-3-APX-R1 | aagaaattcttacacatttaaatGGCTTCAGCAAATCCTAGCTCA |  |
| pLGN-RNAi-3-APX-F2 | agggaattcctgcaggtcgacAGCGACAAGGATATTGTTGC |  |
| pLGN-RNAi-3-APX-R2 | aatttgcaggtatttggatccGGCTTCAGCAAATCCTAGCTCA |  |
| 3APX-RNAi-F | TACCCCACTGTTAGCGAGGATT | Detection of *ClAPX* gene relative expression level by RT-qPCR |
| 3APX-RNAi-R | CTCCGGTCTTGGTCTTCACAT |  |
| CYVCV-614F | TACCGCAGCTATCCATTTCC | Detection of CYVCV by RT-PCR |
| CYVCV-614R | GCAGAAATCCCGAACCACTA |  |
| CYVCV-CP-qF | TCAACCTCAACCTAAGATA | Detection of CYVCV accumulation by RT-qPCR |
| CYVCV-CP-qR | GATGGCGTATAAGAACAC |  |
| Tactin-F | AAGGGATGCGAGGATGGA | Detection of genes in tobacco by RT-qPCR as internal reference |
| Tactin-R | CAAGGAAATCACCGCTTTGG |  |
| CitActin-F | CATCCCTCAGCACCTTCC | Detection of genes in citrus by RT-qPCR as internal reference |
| CitActin-R | CCAACCTTAGCACTTCTCC |  |
| q-CsAOS-F | TCGACGGTGTTTCGTGCTAA | Detection of *CsAOS* gene relative expression level by RT-qPCR |
| q-CsAOS-R | TGCCGTCGAGTAGAACGATG |  |
| q-CsMAPK3-F | TGCTGATCTGGGGTTTGTCC | Detection of *CsMAPK3* gene relative expression level by RT-qPCR |
| q-CsMAPK3-R | ACTTGGGCTAACGACTGACG |  |
| q-CsLOX1-F | GCTGGGGCATATCATTGATGC | Detection of *CsLOX1* gene relative expression level by RT-qPCR |
| q-CsLOX1-R | TTGCACTTGCCCTTTTCACA |  |
| q-CsAOC-F | GAAGGGTGACCGGTTTGAAGC | Detection of *CsAOC* gene relative expression level by RT-qPCR |
| q-CsAOC-R | ACGGCCAAGTACGTGTCTTCG |  |
| q-CsCOI1-F | GGGAATGGAGGATGAAGAAGGT | Detection of *CsCOI1* gene relative expression level by RT-qPCR |
| q-CsCOI1-R | GCCCTGAGCCAAAGCAATTA |  |
| q-CsJAR1-F | AAGGCGATGCAGTCACAATG | Detection of *CsJAR1*gene relative expression level by RT-qPCR |
| q-CsJAR1-R | TGGTGGAAATCAGGACCAAAG |  |
| q-CsLOX3-F | GTCGTTCTGGAACTTGTCGGC | Detection of *CsLOX3* gene relative expression level by RT-qPCR |
| q-CsLOX3-R | CTGTGATTGCACCAGGCGT |  |
| q-CsMYC2-F | GGTGACCATGAGCTCCAACTG | Detection of *CsMYC2* gene relative expression level by RT-qPCR |
| q-CsMYC2-R | GGCCGAAGAGAGATTTGGCTA |  |
| q-CsPDF1.2-F | CAGTGGCAGAAGCAAAACAA | Detection of *CsPDF1.2* gene relative expression level by RT-qPCR |
| q-CsPDF1.2-R | CCGGGGAAGTCGTAGTGGC |  |
| q-CsPR3F | GGCTCAAACTTCACATGAAACTAC | Detection of *CsPR3* gene relative expression level by RT-qPCR |
| q-CsPR3R | GTTGACAATAATCTCCAGGGTTTC |  |
